# Cannabinol for Sleep and Insomnia: A Critical Narrative Review and an Olive-Derived Polyphenol Combination Hypothesis

**DOI:** 10.3390/nu18213659

**Published:** 2026-11-04

**Authors:** Dimitra Pita, Prokopis Magiatis, Evangelos Dadiotis, Eleni Melliou, Ilias Ntoumas, Christina Karatzaferi, Giorgos K. Sakkas

**Affiliations:** 1 Department of Physical Education and Sport Science, University of Thessaly, 42100 Thessaly, Greeceintoumas@uth.gr (I.N.); ck@uth.gr (C.K.); 2 Laboratory of Pharmacognosy and Chemistry of Natural Products, Department of Pharmacy, National and Kapodistrian University of Athens, Panepistimioupoli Zografou, 15771 Athens, Greece; magiatis@pharm.uoa.gr (P.M.); vaggdad@gmail.com (E.D.); emelliou@pharm.uoa.gr (E.M.); 3 Department of Life Sciences, School of Life and Health Sciences, University of Nicosia, 2417 Nicosia, Cyprus

**Keywords:** cannabinol, insomnia, sleep, oleocanthal, oleacein, olive-derived polyphenols, pharmacokinetics, daytime functioning, narrative review

## Abstract

Cannabinol (CBN) is marketed for sleep, but its clinical evidence is limited and heterogeneous. This critical narrative review evaluates CBN-specific sleep studies and considers an olive-derived phenolic combination as a separate, untested clinical hypothesis. Three key randomized trials enrolled different populations and used oral doses of 20–300 mg over one night to four weeks. Primary sleep-quality or sleep-maintenance outcomes were not consistently positive; dose-ranging signals were multiplicity-sensitive and did not identify an optimal dose. At 300 mg, an acute insomnia trial reported selected secondary sleep benefits alongside next-day vigilance and simulated-driving concerns. Rodent findings implicate CBN and its metabolites, but route, exposure, tolerance and dependence limit extrapolation. Oleocanthal studies measured behavioral sleep in disease-model mice, whereas a human olive-phenol trial reported a fatigue benefit rather than a separately demonstrated sleep-domain effect. Parent phenolic instability, metabolism and uncertain target-site exposure weaken direct translation of micromolar assays. The proposed combination warrants exposure-informed investigation, not therapeutic endorsement. Progress requires product-specific safety and analytical qualification, component-controlled experiments and patient-prioritized outcomes alongside established insomnia care.

## 1. Introduction

Insomnia disorder is characterized by persistent difficulty initiating sleep, maintaining sleep or obtaining restorative sleep despite adequate opportunity, together with clinically meaningful daytime impairment. It affects approximately one in ten adults and often follows a chronic or relapsing course, contributing to impaired cognition, mood disturbance, reduced work performance, accidents and increased health-care use [1,2]. Cognitive behavioral therapy for insomnia (CBT-I) remains the recommended first-line treatment, while pharmacotherapy is generally reserved for selected patients, limited periods, or situations in which CBT-I is unavailable or insufficient [1]. This therapeutic context is important: any cannabinoid-based product should be evaluated as an experimental or adjunctive intervention rather than positioned as a substitute for evidence-based behavioral care.

Interest in cannabinol (CBN), a minor phytocannabinoid, has grown alongside the use of cannabis-derived sleep products. It must be distinguished from cannabidiol (CBD) and Δ9-tetrahydrocannabinol (THC): evidence for one constituent, or a mixed preparation, cannot be assigned to another. The earlier reputation of CBN as a sleep aid substantially preceded controlled CBN-specific evidence [3,4,5,6,7].

Olive-derived phenolics provide a possible research extension, not an established partner therapy. The rationale combines three observations: CBN can alter sleep-related physiology; oleocanthal has affected behaviourally estimated sleep in mice; and cannabinoid–olive-phenolic interactions have been reported in non-sleep experimental systems. A human secoiridoid-rich supplementation study also reported reduced fatigue. These observations justify asking a question about combined activity, but they differ in compounds, exposure, model and endpoint [8,9,10,11,12,13,14].

Two olive-related questions must remain separate. Olive oil is a lipid delivery vehicle that may modify cannabinoid exposure. Added phenolics—such as oleocanthal, oleacein, hydroxytyrosol and related secoiridoids—are potentially active chemical constituents. Improved delivery, an additional biological effect and an interaction between components require different comparisons. This review therefore centers on CBN and sleep; the olive-phenolic combination is explicitly presented as a hypothesis. It is neither a clinical-trial protocol nor an endorsement of a particular product.

## 2. Methods of the Narrative Review

This critical narrative review uses the reporting domains of the Scale for the Assessment of Narrative Review Articles (SANRA) to clarify its aim, search, source selection and scientific reasoning [15]. The 64 references in the submitted manuscript were the defined seed set. Its documented update on 27 October 2026 included co-author-nominated publications from the authors’ own research groups, including OleoMetS and conference/patent material. These sources were not treated as independent replications.

For the revision, targeted source retrieval and verification were conducted on 27 October 2026. PubMed-indexed records, PubMed Central and publisher full-text pages were retrieved through domain-focused web searches, supplemented by exact-title/DOI checks, backward reference checking, Google Patents, and official regulatory websites. Searches combined cannabinol/CBN and sleep/insomnia with oleocanthal, oleacein, olive phenolics, hydroxytyrosol, pharmacokinetics, bioavailability, tolerance, melatonin, transient receptor potential ankyrin 1 (TRPA1) and combination/interactions. There was no earliest-publication restriction; the revision cutoff was 27 October 2026.

Selection prioritized primary CBN human interventions with sleep endpoints, relevant animal sleep and safety studies, compound-specific exposure studies, and molecular reports addressing the proposed combination. Positive, negative, and safety findings were considered. Mixed-cannabinoid trials, observational dietary studies and non-sleep models were retained only as contextual or indirect evidence. Conference abstracts and patent disclosures were labeled separately from full peer-reviewed reports; unpublished laboratory information was not used to fill reporting gaps. English-language reports or records with interpretable English content were assessed. Unrelated applications, marketing claims and duplicate versions were excluded from the clinical efficacy synthesis. No pooled effect, formal risk-of-bias score, independent duplicate screening or exhaustive-retrieval claim is made.

## 3. Clinical Context and Patient-Relevant Outcomes

Sleep difficulties in a symptom-selected population are not synonymous with diagnosed insomnia disorder. The Insomnia Severity Index (ISI) measures perceived symptom burden; a formal diagnosis also requires clinical assessment. Polysomnography (PSG) measures electroencephalographic (EEG) sleep stages and continuity, whereas actigraphy estimates sleep from movement. Neither an improved questionnaire score nor more time scored as sleep necessarily means improved daytime functioning [1,3,16,17].

For a prospective user, next-day alertness, safe driving, ability to work, falls, cognition and freedom from tolerance or dependence are central—not optional additions to sleep-onset latency. Sleep quality, fatigue and daytime-function outcomes should be measured and analyzed separately. Patient-reported outcomes and objective sleep measures answer complementary questions; disagreement between them is itself clinically relevant [1,16,17].

The review’s clinical interpretation is therefore based on benefit–harm balance and care pathways. Cognitive behavioral therapy for insomnia (CBT-I) should remain available rather than be displaced by self-medication. Future research could test an investigational intervention alongside stable usual care or as an adjunct to CBT-I, with access, adherence and treatment changes recorded. People with insomnia should help select outcomes, acceptable next-day effects, treatment burden and follow-up duration before such a trial is finalized; this is a recommendation for future involvement, not a claim that patient involvement occurred in this review [1].

## 4. CBN: Pharmacology, Exposure and Preclinical Evidence

### 4.1. Chemical Identity and Receptor Activity

CBN is a minor phytocannabinoid that arises principally through oxidative transformation of THC during plant aging, processing and storage. Its concentration in a preparation can therefore reflect both plant chemistry and product degradation. CBN is highly lipophilic and poorly water soluble, characteristics that complicate oral delivery and may produce variable absorption. Reviews of its chemistry emphasize that CBN should not be regarded merely as an inert marker of old cannabis: it has pharmacological activity, but its receptor profile, metabolites and dose–response relationships remain less fully characterized than those of THC or CBD [6].

Cannabinoid receptor type 1 (CB1) and type 2 (CB2) signaling is assay dependent. Arnold et al. found low partial agonist activity of CBN at human CB1 receptors (Emax 29 ± 3%) and substantially greater partial agonist activity of 11-hydroxy-CBN (11-OH-CBN; Emax 75 ± 3%), with the metabolite approaching Δ9-THC in potency and efficacy in their membrane-potential assay [14]. In contrast, Kundu et al. reported no CB1 activation by CBN or CBN-11-OH in their receptor-activation assays; CBN and several metabolites, including CBN-11-OH, instead antagonized CB1/CB2, whereas CBN-1′-OH showed partial CB1 agonism [18]. These discordant findings should be retained rather than harmonized, because assay platform, receptor expression, readout and concentration can materially alter apparent cannabinoid pharmacology. Transient receptor potential ankyrin 1 (TRPA1) is another molecular target, but activation of an expressed ion channel does not specify a net effect on human sleep [19].

### 4.2. Pharmacokinetics and the Active-Metabolite Hypothesis

The rat sleep study used intraperitoneal, rather than oral, administration. It measured CBN and 11-OH-CBN in plasma and brain and found that the metabolite could independently alter sleep. Those observations motivate metabolite measurement, but do not show that oral human dosing produces the same parent-to-metabolite ratio or brain concentrations. The amount and duration of human exposure must be characterized directly. A reliable formulation-independent estimate of absolute oral CBN bioavailability cannot be assigned from the studies synthesized here [14,17,18,20].

The distinction matters because dissolution, food, first-pass metabolism and active metabolites can change both efficacy and next-day risk. An oral product comparison needs matched dose and meal conditions and measurement of parent CBN and relevant metabolites; nominal milligrams alone are an incomplete exposure measure. Results for oral CBD cannot be used as numerical CBN bioavailability estimates. They provide a formulation precedent only [21,22].

### 4.3. Metabolism and Interaction Potential

In vitro cytochrome P450 studies support the possibility of cannabinoid–medicine interactions, but enzyme inhibition is not a patient-level interaction estimate [21]. Of direct relevance to sleep products, CBN inhibited cytochrome P450 1A2 (CYP1A2)-mediated melatonin metabolism in vitro and increased melatonin exposure approximately fourfold in mice [23]. That is a preclinical interaction signal, not a demonstrated fourfold effect in humans. CBN–melatonin co-use should therefore not be assumed equivalent to either ingredient alone, and endogenous melatonin effects remain a separate question. Human findings with other CYP1A2 inhibitors are contextual rather than CBN evidence [24].

Medication review should consider other sedatives, alcohol and drugs with narrow therapeutic indices, with decisions based on the actual formulation, expected exposure and individual treatment rather than an unqualified class-wide prohibition. The clinically important concerns are altered drug exposure and additive impairment. Dedicated interaction studies and pharmacovigilance are needed before reassuring patients who use several medicines [21,23].

### 4.4. Sleep Architecture, Tolerance and Dependence in Animals

Arnold et al. administered CBN at 10, 30, or 100 mg/kg intraperitoneally to male rats and separately tested 11-OH-CBN; repeated CBN was studied at 10 mg/kg/day for 15 days. The authors reported 100% CBN purity with an in-house analytical check. EEG/electromyographic (EMG) recordings showed time-dependent changes in non-rapid-eye-movement (NREM) and rapid-eye-movement (REM) sleep, with early suppression followed by later increases and partial tolerance to repeated dosing. These findings favor a time-resolved rather than uniformly sedative interpretation [14].

Vanegas et al. reported tolerance and antagonist-precipitated withdrawal after high-dose CBN exposure in mice, including 100 mg/kg twice daily for five days. CBN also fully substituted for THC in a drug-discrimination task, indicating THC-like interoceptive stimulus effects in that animal model. This is not a direct measurement of human subjective intoxication, but it makes a categorical “non-psychoactive” or dependence-free description inappropriate [25]. The separate TruCBN™ toxicology program requires similarly qualified interpretation. Although the definitive 90-day rat study established a no-observed-adverse-effect level of 400 mg/kg/day, post-dose hypoactivity and reduced male body weight/food consumption were observed mainly at 200–400 mg/kg/day, and the preceding range-finding program produced mortality or humane sacrifice at 1750 and 3500 mg/kg/day [26]. The study was funded by FloraWorks, the manufacturer of TruCBN™, and four authors were affiliated with FloraWorks; the article states that the work was conducted in the normal course of employment. These industry links do not invalidate the data, but they are relevant to interpretation alongside the product-, species-, and endpoint-specific nature of the findings.

Older anesthetic or barbiturate sleeping-time experiments gave inconsistent results for CBN alone and interactions with THC. Such assays can reflect altered metabolism or generalized depression rather than physiological sleep [27,28,29]. Together, these data support additional exposure-aware work and prospective tolerance/withdrawal assessment, not a presumed safety advantage of chronic CBN use.

## 5. Human CBN Evidence

Three key randomized CBN-containing sleep trials are considered below [16,17,30]. They differ in diagnostic status, dose, formulation, duration, comparator, and endpoint. Meaningful comparison is therefore qualitative: each treatment is interpreted against its own randomized control and primary endpoint. Their sample sizes cannot be summed as three replications in diagnosed insomnia, and statistical significance across different studies cannot rank doses. Table 1 presents the main results and limitations together.

### 5.1. Seven-Night Trial in People Reporting Poor Sleep

Bonn-Miller et al. randomized 321 participants; 293 consumed study product and entered the modified intention-to-treat analysis [16]. The trial planned approximately 375 participants (75 per arm), allowing for 20% attrition, but recruitment was terminated early because of slowed recruitment and limited time. The sample-size calculation targeted 80% power to detect an odds ratio of 3.0 and used a two-sided Bonferroni-corrected α of 0.0125 for four comparisons with placebo. The primary sleep-quality contrast for 20 mg CBN was inconclusive (odds ratio 2.26; 95% confidence interval 0.93–5.52; *p* = 0.082). Nominal benefits in reported awakenings and overall sleep disturbance were secondary; sleep-onset latency, wake after sleep onset (WASO), and daytime fatigue did not clearly differ from placebo. Adding CBD did not strengthen the effect. The under-recruited sample is therefore relevant when interpreting the negative primary endpoint, but it does not convert secondary findings into confirmatory evidence.

The Patient-Reported Outcomes Measurement Information System (PROMIS) Sleep Disturbance measure captures the participant’s perceived sleep difficulties. It is useful for the patient experience, but is neither a diagnostic interview nor an EEG measure of sleep architecture. This distinction also applies to the decentralized dose-ranging trial [16,30].

### 5.2. Four-Week Dose-Ranging Trial: Small, Non-Ranked Signals

Kolobaric et al. randomized 1020 adults to 25, 50 or 100 mg CBN, 4 mg melatonin, or placebo [30]. The reported placebo-relative arm-by-week coefficients were similar: −0.544, −0.603 and −0.566 for the three CBN doses and −0.564 for melatonin. These are trajectory coefficients, not direct between-dose effects. The study did not establish that 50 mg was preferable or that a nonlinear dose–response relationship explained the findings.

The placebo-relative *p* values of approximately 0.018–0.030 are nominal and would not survive a conservative Bonferroni correction across the three CBN–placebo contrasts. The report describes Bonferroni-adjusted post hoc comparisons; its nominal longitudinal signals should not be presented as robust multiplicity-adjusted confirmation. Clinically meaningful responder comparisons did not clearly distinguish CBN from placebo, and no CBN dose differed significantly from melatonin. Absence of a difference is not equivalence, and the similar active-arm trajectories do not identify their mechanism. Approximately one quarter of randomized participants contributed no follow-up data, increasing sensitivity to assumptions about missingness [30].

### 5.3. Acute Trial in Diagnosed Insomnia: Sleep and Next-Day Effects

The CUPID crossover study enrolled 20 adults with diagnosed insomnia and tested 30 mg, 300 mg, and placebo on separate nights [17,20]. The primary PSG WASO contrasts were −4.0 min (95% confidence interval −15.9 to 7.9) at 30 mg and −6.3 min (−18.2 to 5.5) at 300 mg. Selected secondary measures, including sleep-onset latency, subjective sleep quality, stage N2 sleep and arousal indices, favored 300 mg. The negative primary result and multiplicity require these findings to remain endpoint-specific.

Next-day assessment is particularly important: at 300 mg, slower performance on the psychomotor vigilance task (PVT) and greater simulated-driving speed variability were reported. These task-specific findings are not proof that every driving measure worsened, but they materially qualify the sleep results. Mild adverse events numbered 91 at 300 mg, 73 at 30 mg and 82 on placebo; the single moderate event occurred on placebo. The aggregate total of 247 events should therefore not be used as evidence of a large drug-attributable adverse-event burden. Conversely, the small, acute study cannot exclude uncommon or cumulative harms [17].

### 5.4. Overall Clinical Interpretation

The evidence indicates possible effects on selected aspects of sleep, but no reproducible dose hierarchy, established long-term benefit, or demonstrated freedom from next-day impairment. The three trials should not be characterized as consistently positive studies of insomnia treatment. Mixed-cannabinoid trials and broader meta-analyses may inform context, but their pooled estimates do not isolate CBN or validate an olive-phenolic partner [3,5,16,17,20,30,31,32,33].

## 6. Olive Phenolics: Sleep Signals, Human Exposure and Dose Translation

### 6.1. Composition and Non-Interchangeable Preparations

Extra-virgin olive oil, a secoiridoid-rich extract, isolated oleocanthal, isolated oleacein and a hydroxytyrosol-standardized supplement are different interventions. Relevant constituents include oleocanthal, oleacein, oleuropein and ligstroside aglycones, hydroxytyrosol and tyrosol. Their relative abundance varies with production and storage. A total-phenol assay or the concentration of one marker cannot characterize all biological exposure; an 80% mixed fraction is a composition statement, not 80% purity of each constituent [8,34].

### 6.2. Dietary and Preclinical Evidence

Associations between Mediterranean dietary patterns or dietary polyphenols and reported sleep quality remain subject to dietary, behavioral and socioeconomic confounding and reverse causality [35,36,37,38]. They should not be used to attribute a sleep benefit to a particular olive compound. Similarly, the European olive-polyphenol health claim concerns protection of blood lipids from oxidative stress under defined conditions, not a sleep indication or an effective insomnia dose [39].

Yang et al. administered oleocanthal at 10 mg/kg/day orally for three months to male Alzheimer’s disease-model mice and observed age-dependent changes in sleep-related behavior [9]. A subsequent 0.5–20 mg/kg/day study reported dose-dependent metabolic/sleep-related findings and liver toxicity at 20 mg/kg [10]. Sleep was inferred from cage inactivity, including a threshold exceeding 40 s, rather than EEG-defined stages. Reduced movement, disease-model effects and single-sex sampling limit translation. These are oleocanthal observations, not interchangeable evidence for oleacein or CBN combinations.

Hydroxytyrosol butyrate reduced selected inflammatory or mitochondrial consequences of experimentally imposed sleep loss in a separate model [40]. Protection from sleep-loss injury is distinct from inducing physiological sleep. The derivative, experimental context and endpoint must remain identifiable in the interpretation.

### 6.3. Human Supplementation: OleoMetS

OleoMetS is the randomized olive-oil-phenol supplementation study in adults with metabolic syndrome reported by Samoutis et al. [8]. It enrolled 102 participants for 12 weeks; the daily 10 mg extract comprised approximately 80% combined oleocanthal/oleacein and 18% oleuropein/ligstroside aglycones. The main trial questions were metabolic. The methods describe an Energy Level Questionnaire and cite the Multidimensional Fatigue Inventory (MFI), whose validated domains are general fatigue, physical fatigue, mental fatigue, reduced activity and reduced motivation; the MFI has no sleep domain. Although the OleoMetS baseline table also labels a “Sleep Quality Score,” the manuscript does not define or validate a separate sleep scale or report a sleep-specific treatment contrast. The overall ELQ score worsened by +6.94 points in the control arm and improved by −9.94 points in the intervention arm, yielding the reported 16.88-point between-group difference (*p* < 0.0001). Thus, part of the contrast reflects deterioration in the control group. We therefore treat OleoMetS as evidence of a fatigue/daytime-function signal from a characterized olive-phenolic preparation, not as evidence that the preparation improves sleep.

### 6.4. Bioavailability: The Chemical Species Reaching the Circulation

A major translational obstacle is that the ingested parent phenolics may not be the dominant circulating species. Gastric hydrolysis and subsequent metabolism can generate hydroxytyrosol, tyrosol and conjugates [34]. The OleoMetS report discusses the lack of directly measurable parent oleocanthal/oleacein in human plasma and measurable amino-acid adducts, including oleoglycine, with an early peak around 30 min [8]. Such observations depend on sampling and assay sensitivity; nondetection is not proof of zero transient or local exposure, and an oleocanthal-derived adduct should not automatically be assigned to oleacein.

Nevertheless, an assay exposing cells continuously to micromolar parent compounds may test a chemical environment unlike that after oral use. Total plasma concentration, unbound concentration and brain exposure are different quantities. Future exposure studies should identify the parents, stable adducts and relevant metabolites in the actual product and biological samples, establish their stability during collection, and distinguish true in vivo conversion from ex vivo reaction. Until then, micromolar synergy claims are mechanistic leads rather than evidence of attainable human target engagement.

### 6.5. Dose Translation and Its Limitations

Using the conventional body-surface-area conversion (mouse Km = 3; adult Km = 37), 10 and 20 mg/kg/day in mice correspond to approximately 0.81 and 1.62 mg/kg/day, or 57 and 114 mg/day for a 70 kg adult [41]. These are calculated human-equivalent amounts, not recommended or safe human doses. They do not account for route-dependent exposure, metabolites, disease state or a safety factor. The 20 mg/kg mouse dose associated with hepatic toxicity is not a no-adverse-effect reference dose [10].

For scale, OleoMetS supplied approximately 8 mg/day of oleocanthal plus oleacein combined, not 8 mg of oleocanthal alone [8]. A commonly cited dietary estimate is around 9 mg/day of oleocanthal under particular intake and composition assumptions; it is not a universal dietary exposure [42]. The larger animal-equivalent amounts cannot be used to infer a human therapeutic window. Human toxicity at comparable chronic oleocanthal exposures and repeated-dose safety of the intended CBN–phenolic combination remain unresolved; appropriate nonclinical support must precede such exposures [43].

## 7. The Olive-Phenolic Combination Hypothesis: Evidence and Discriminating Tests

The remaining question is whether a defined phenolic preparation adds benefit to CBN at tolerable, attainable exposure. New animal studies, human supplementation results, and preliminary molecular observations justify investigation, but they do not collectively constitute a sleep-combination trial. No sleep-focused intervention directly testing CBN with a defined olive-derived phenolic fraction was identified in the sources examined. The mechanisms below are ranked as observed findings, exposure-dependent inferences or proposed experiments, rather than assembled into an assumed beneficial pathway.

### 7.1. Lipid Delivery Is a Separate Hypothesis

Olive oil can act as a cannabinoid extraction and delivery matrix, but formulation studies show carrier-dependent differences and do not establish CBN-specific superiority over other oils [44]. Zgair et al. found that oral lipid co-administration increased rat exposure to THC and CBD approximately 2.5- and 3-fold, respectively, and investigated intestinal solubilization and chylomicron association [22]. This is an appropriate primary formulation precedent, not evidence that olive phenolics increase human CBN absorption. A carrier comparison must hold CBN dose and phenolic content constant; a phenolic comparison should use the same characterized carrier.

### 7.2. Cannabinoid Signaling and Competing Biological Explanations

Endocannabinoid experiments support a role for CB1 signaling in sleep–wake regulation, but receptor agonism alone does not specify a sustained therapeutic effect [45,46,47,48]. The CBN/11-OH-CBN rat work provides a stronger sleep-specific link than most proposed olive pathways [14]. Olive-related changes in cannabinoid receptor expression have been reported in intestinal cells and animal intestinal/cancer models [49,50]. They do not show increased central CB1 availability or prevention of CBN tolerance. The latter proposal should be tested by measuring central receptor expression/function, oral exposure, and repeated-dose sleep effects, not inferred from intestinal messenger RNA.

Oleacein has been reported to act as a tropomyosin receptor kinase B (TrkB) agonist and to increase brain-derived neurotrophic factor (BDNF). The evidence includes surface-plasmon-resonance binding and blockade of BDNF induction by pathway inhibitors, without a direct TrkB-phosphorylation demonstration. The paper includes both an acute oral reporter experiment and a separate 10-day, 10 mg/kg/day mouse inflammation experiment—not only a single in vivo exposure [51]. These data concern neurotrophic/inflammatory responses rather than sleep. The connection to slow-wave activity through BDNF is therefore indirect [52].

Other candidate targets may predict opposing effects. Peroxisome proliferator-activated receptor alpha (PPARα) activation has wake-promoting evidence, while cyclooxygenase (COX) inhibition may alter both sleep-promoting prostaglandin D2 and wake-promoting prostaglandin E2. Neither a net COX-related sleep benefit nor a universal arousal effect follows from those pathways [42,53,54,55,56,57]. These alternatives should be retained as competing explanations, not counted as independent support for the same desired outcome.

### 7.3. TRPA1: Context, Combination Records and Author-Linked Evidence

TRPA1 activation can have different consequences in different tissues and states. Irritant-evoked arousal and oleocanthal pungency support sensory activation pathways [58,59]. In a REM-sleep-deprivation rat model, an intrathecal TRPA1 antagonist (Chembridge-5861528) attenuated mechanical hypersensitivity [60]. This is a pain outcome after sleep loss, not evidence that the antagonist improves sleep. Findings in airway and temperature-regulation models further caution against assigning one direction to all TRPA1 activity [61,62,63].

The conference communications by Dadiotis and colleagues originate from laboratories represented among this review’s authors [11,12]. They report cannabinoid–secoiridoid interactions, including CBD–oleocanthal modulation of TRPA1. These remain abstract-level reports; underlying datasets and any methods beyond those publicly disclosed have not been incorporated as unpublished evidence in this review. The single-dose mouse observation cited in the conference material does not provide a sufficiently specified cannabinoid identity/ratio and a repeated-exposure design to support a chronic CBN-combination safety claim.

The published patent application WO2025219345A1, for which co-author Evangelos Dadiotis is a named inventor and Ekati Biotech PC is the listed applicant, contains non-sleep cannabinoid–olive-phenolic experiments [13]. Its TRPA1 calcium-mobilization experiments include CBN with oleocanthal or oleacein in transfected HEK293E cells; the described assay follows acute addition over approximately 150 s. Patent composition ratios must not be treated as the tested concentrations of every experiment.

Importantly, the hippocampal hyperexcitability experiment concerns CBD, not CBN. The patent describes a 1:8 CBD–oleocanthal combination that suppressed high-potassium-induced spontaneous activity, with attenuation of that effect by HC-030031 [13]; the related abstract also addresses CBD [12]. This observation is compatible with a context-dependent inhibitory network effect despite TRPA1 activation. It is neither a sleep endpoint nor proof of the same action after oral CBN. The distinction between acute molecular assays, non-sleep animal observations and sleep-specific clinical evidence is retained throughout.

### 7.4. A Directional Prediction That Can Be Falsified

Our proposed, explicitly unconfirmed clinical prediction is that a phenolic addition might reduce nocturnal fragmentation or its daytime consequences beyond CBN alone without increasing residual impairment. Its basis is the combination of CBN-related sleep effects, oleocanthal behavioral-sleep observations, and context-dependent network/inflammatory findings—not a claim that TRPA1 agonism is uniformly sedative. Equally plausible alternatives are no added effect at human exposure, exposure-mediated CBN potentiation with worse next-day performance, or increased sensory arousal.

**Figure 1:**
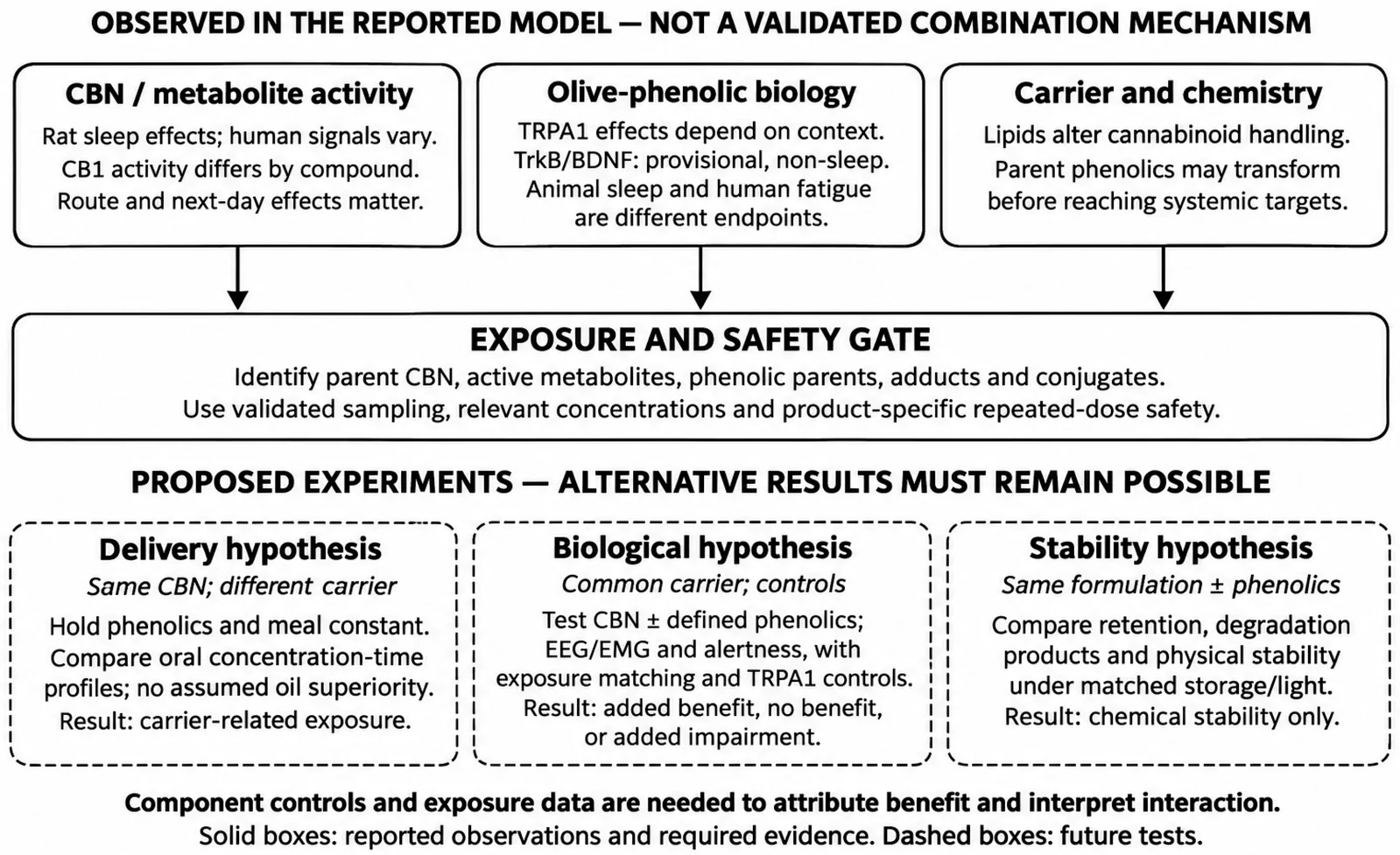
Evidence-informed hypotheses and experiments that can distinguish them. Solid panels identify findings within their reported models; the lower panels describe proposed experiments, not completed studies. The carrier comparison holds phenolic exposure constant, whereas the phenolic comparison uses a common carrier. CBN, cannabinol; CB1, cannabinoid receptor type 1; TRPA1, transient receptor potential ankyrin 1; EEG, electroencephalography; EMG, electromyography; TrkB, tropomyosin receptor kinase B; BDNF, brain-derived neurotrophic factor. Sources: [8,9,10,11,12,13,14,18,19,21,22,44,45,46,47,48,49,50,51,58,59,60].

A discriminating sequence would first test CBN—not substitute CBD—with the intended phenolics and their measurable metabolites at exposure-relevant concentrations, including vehicle, individual components and combinations. Concentration–response matrices and an explicit additivity model would separate additivity from interactions. Oral, repeated-dose EEG/EMG experiments should then combine matched exposure measurements with TRPA1 blockade or appropriate genetic controls, and assess both sleep and next-period alertness. Controls receiving the blocker alone are essential because pathway manipulation may itself affect sleep. A later component-controlled human comparison would assess the prediction clinically. If an apparent added effect disappears after CBN-exposure matching, a pharmacokinetic explanation is favored; persistent receptor-dependent effects would support further pharmacodynamic investigation (Figure 1).

## 8. Formulation, Purity and Analytical Requirements

### 8.1. Composition and Comparability

An isolate’s stated chemical purity, the amount of active compound per dose, an extract’s relative composition and a finished product’s contaminant limits are different measurements. The rat CBN study reports 100% purity with analytical assessment [14], whereas OleoMetS specifies the relative composition of a mixed phenolic preparation [8].

For a combined investigational product, identity and quantity should cover CBN and the phenolics actually present, including oleocanthal, oleacein, relevant aglycones, hydroxytyrosol and tyrosol. Cannabinoid impurities and transformation products, particularly THC where relevant, require adequate specificity. Published nuclear magnetic resonance, chromatographic and mass-spectrometric methods provide useful starting points, but extraction recovery and interference must be assessed in the final matrix [64,65,66,67].

### 8.2. Proportionate Analytical and Stability Requirements

Analytical requirements should be proportionate to the formulation and development stage rather than presented as a universal product-development checklist. For medicinal-product work, fit-for-purpose methods can draw on ICH Q2(R2), Q14 and M10 principles for identity, potency, relevant impurities, dose uniformity and biological-sample measurement [68,69,70]. Which contaminants or matrix-specific tests are needed should follow from the actual raw materials, extraction process, excipients and route of administration.

Stability likewise requires product-specific evidence. Carrier-oil studies show that composition and storage can alter cannabinoid quality, while olive secoiridoids are themselves chemically labile and can hydrate or react with primary amines [44,64,71,72]. Appropriate real-time, stress or light-exposure studies can therefore test whether the proposed combination remains chemically defined; ICH Q1A(R2) and Q1B provide relevant stability principles without implying that a review article can prescribe a full regulatory dossier [73,74].

## 9. Safety, Current Use and Regulatory Context

### 9.1. Safety and Current-Use Considerations

CBN-containing products, including products combined with melatonin, are marketed as sleep aids in some jurisdictions, but availability does not establish an approved insomnia indication [7,23,75,76]. Current evidence supports caution about next-day impairment, the preclinical CBN–melatonin interaction, tolerance/dependence at high animal exposures and the limitations of short-duration human studies [17,21,23,25,26]. Older adults and people taking multiple medicines warrant particular attention because falls, cognitive vulnerability and altered drug exposure may change the benefit–harm balance. Investigational use should not displace evaluation of other sleep disorders or access to cognitive behavioral therapy for insomnia.

Concentrated olive phenolics and CBN–phenolic combinations also require exposure-based, product-specific safety assessment rather than extrapolation from dietary olive oil or from CBN alone. The oleocanthal liver signal and the TruCBN range-finding findings illustrate why dose, duration and formulation matter [10,26].

### 9.2. Proportionate Regulatory Context

The regulatory pathway depends on the actual product and intended claim. ICH M3(R2) provides a framework for deciding what nonclinical support is appropriate to route, dose, duration, and existing knowledge [43]. If the combination is studied as a medicinal product in the European Union, Regulation (EU) No 536/2014, CTIS and ICH E6(R3) are relevant [77,78]. Consumer-food or cannabis frameworks are jurisdiction-specific and do not confer a therapeutic indication merely because a product is commercially available [75,76,79,80].

Accordingly, public registration, ethics review, clinical-trial authorization and marketing permission should remain conceptually separate. The purpose of this review is to identify the safety and evidentiary gaps that precede clinical translation, not to specify a development dossier.

## 10. Research Priorities Arising from the Evidence Gaps

Table 2 summarizes what has been observed and what remains unresolved. The following priorities are conclusions from the evidence appraisal, not a proposed protocol with a fixed dose, sample size or treatment schedule.

### 10.1. Resolve Exposure Before Inferring Target Engagement

The most consequential missing link is exposure to the actual active species. Research should measure parent CBN, 11-OH-CBN and other relevant CBN metabolites together with oleocanthal, oleacein, oleoglycine and other validated adducts, hydroxytyrosol/tyrosol and relevant conjugates. Animal data already indicate extensive presystemic metabolism: after oral intake in rats, oleocanthal itself was detected only in the stomach and intestine, whereas its phase I and II metabolites were distributed to tissues [81], and after intraperitoneal dosing in mice the parent compound was not detected in plasma, with oleocanthalic acid proposed as an exposure biomarker [82]. Dense early sampling is justified by rapid phenolic transformation, while later sampling should capture CBN persistence and next-day exposure. Total and unbound concentrations, meal conditions, and ex vivo stability require attention. A measurement panel must be technically validated rather than assume that every proposed species can already be quantified [8,14,18,23,34,70].

### 10.2. Establish Product-Specific Safety and Component Contribution

Progression to a sleep-efficacy trial depends on an adequate formulation and safety dossier, including repeated-dose combination information appropriate to the intended clinical exposure [43,68,69,70,73,74,77,78]. The evidence does not justify choosing 50 mg CBN because of a purported optimal-dose result, or extrapolating single-night 300 mg findings to long-term combination use. Candidate doses should be justified by measured exposure, safety and a prospectively defined clinical question, not by selecting favorable secondary endpoints.

A combination–placebo comparison estimates the effect of the complete product. Attribution to the phenolics requires at least a CBN-alone comparator; a component-controlled factorial design can additionally estimate an interaction under a prespecified scale/model. Such a design must be powered for its actual contrast and interpreted with exposure data. Statistical interaction is not synonymous with molecular synergy. The matched carrier should be held constant unless a separate carrier effect is the intended question.

### 10.3. Put Clinical Utility and Patient Involvement First

People with sleep difficulties and diagnosed insomnia should help prioritize the magnitude of benefit worth pursuing, acceptable residual effects, follow-up, and participant burden. The studied population and usual-care pathway must be explicit. Meaningful outcomes include symptom burden, objective continuity, daytime functioning, driving-related performance and tolerability; fatigue and sleep subscales should not be merged into one efficacy claim. Trials should be prospectively registered with a statistical plan, multiplicity control and transparent handling of discontinuation and missing observations. Negative, null and adverse findings are informative outcomes of the research agenda [1,16,17,30,77,78].

## 11. Limitations

This is a targeted critical narrative synthesis. The seed bibliography and executed revision-stage retrieval are documented, but there was no prospectively specified systematic-review protocol, exhaustive database export, independent duplicate screening or formal risk-of-bias assessment. Several reports originate from laboratories represented among the authors. Evangelos Dadiotis is an inventor on WO2025219345A1, while Prokopis Magiatis and Eleni Melliou are inventors on the WO2020165614 patent family relevant to olive-secoiridoid extraction. These relationships are disclosed rather than treated as evidence of independent confirmation.

Some trial materials, conference details, batch-purity information and numerical assay specifications were not independently recoverable from the accessible sources; these gaps are explicitly marked in the Supplementary Materials rather than imputed. The OleoMetS methods cite the MFI, which has no sleep domain, despite a baseline table entry labeled “Sleep Quality Score”; because no validated sleep measure or sleep-specific treatment contrast is defined, this review does not use OleoMetS as sleep-efficacy evidence. Comparisons remain sensitive to population, formulation, route, duration, endpoint and industry sponsorship. These limitations preclude treatment recommendations or an assurance that all relevant combination studies have been retrieved.

## 12. Principal Unresolved Questions

The central uncertainties are whether CBN offers a clinically worthwhile sleep benefit without next-day cost; which parent and metabolite exposures drive that balance; whether effects persist or show tolerance; and whether a defined olive-phenolic fraction adds benefit at attainable exposure. The answers may differ in symptom-selected adults, diagnosed insomnia, and older people with polypharmacy. For the combination, physiological sleep outcomes and repeated-dose safety are more important next steps than further accumulation of unrelated antioxidant or assay-level claims.

## 13. Conclusions

CBN has experimental sleep activity, but its human evidence remains heterogeneous, short-term and endpoint-specific. The available trials do not identify an optimal dose, and the acute 300 mg findings require explicit consideration of next-day vigilance and driving-related performance. Olive phenolics add animal sleep observations, human fatigue evidence and preliminary non-sleep molecular interactions, but these sources address different questions.

The olive-derived phenolic combination is an exposure-dependent hypothesis, not a demonstrated sleep therapy. Advancement is scientifically reasonable only through product-specific analytical and safety qualification, confirmation of attainable active species, and patient-centered testing alongside established care. Component-controlled comparisons are needed for attribution and interaction; a benefit from a complete product alone cannot establish synergy. This evidence-based distinction is the principal conclusion of the review.

## Figures and Tables

**Table 1:** Key randomized CBN sleep studies: population, within-study results and interpretive limits.

Study/Population	Exposure and Design	Primary/Principal Result	Secondary and Safety Findings	Interpretive Limits
Bonn-Miller et al. [16] 321 randomized; 293 in modified intention-to-treat analysis; poor self-rated sleep, not a required insomnia diagnosis.	20 mg CBN alone or +10/20/100 mg CBD; placebo; 7 nights.	Sleep-quality primary outcome: OR 2.26 (95% CI 0.93–5.52), *p* = 0.082 for CBN alone.	Nominal reductions in awakenings (*p* = 0.025) and sleep disturbance (*p* = 0.023); no clear sleep-onset, WASO or fatigue benefit.	Short exposure; subjective measures; 321 randomized versus a planned target of 375 (75/arm); recruitment stopped early because of slowed recruitment and limited time. Power calculation used Bonferroni-corrected α = 0.0125. Funded by Canopy Growth Corporation.
Kolobaric et al. [30] 1020 randomized; general sleep-improvement population.	CBN 25/50/100 mg, melatonin 4 mg or placebo; 4 weeks.	Placebo-relative arm-by-week coefficients: −0.544/−0.603/−0.566; melatonin −0.564. Nominal *p* approximately 0.018–0.030.	Meaningful-response comparisons did not clearly separate from placebo. No CBN dose was shown superior to melatonin.	Multiplicity-sensitive signals; about one quarter lacked follow-up. No formal evidence of an optimal CBN dose. Study conducted by Radicle Science under contract with, and funded by, FloraWorks.
Lavender et al. [17] 20 adults with diagnosed insomnia.	30/300 mg CBN or placebo; single-night crossover with PSG.	WASO: 300 mg −6.3 min (95% CI −18.2 to 5.5); 30 mg −4.0 min (−15.9 to 7.9). Neither significant.	300 mg altered selected secondary sleep measures; slower PVT and greater driving-speed variability. Mild events: 91/73/82 for 300/30/placebo; one moderate event on placebo.	Acute, small sample; multiple outcomes; next-day findings relevant despite an inconclusive primary sleep endpoint.

CBN, cannabinol; CBD, cannabidiol; CI, confidence interval; OR, odds ratio; PSG, polysomnography; PVT, psychomotor vigilance task; WASO, wake after sleep onset. Coefficients in the Kolobaric study describe outcome trajectories, not direct dose comparisons. Nominal significance is not multiplicity-adjusted evidence. Adverse-event counts are events, not numbers of affected participants. Industry context: Bonn-Miller et al. [16] was funded by Canopy Growth Corporation; Kolobaric et al. [30] was funded by FloraWorks and conducted by Radicle Science under contract; the related TruCBN™ toxicology study [26] was also funded by FloraWorks.

**Table 2:** Evidence map distinguishing clinical sleep, non-sleep clinical, preclinical, molecular, and formulation findings.

Evidence Category	Observed Result/Exposure	Sleep Relevance and Limitation	Priority That Follows
Human CBN sleep trials [16,17,30]	20–300 mg; 7-night, 4-week or acute crossover exposure. Primary findings and numerical estimates in Table 1.	Two symptom-selected cohorts; one diagnosed-insomnia cohort. No optimal dose; next-day effects matter.	Replicate a prespecified patient-relevant effect with exposure and impairment assessment.
Human olive-phenol supplementation: OleoMetS [8]	102 adults; 12 weeks; 10 mg extract/day, ~8 mg combined oleocanthal/oleacein. ELQ overall change: control +6.94 (worsening), intervention −9.94 (improvement); between-group difference 16.88 points, *p* < 0.0001.	Metabolic-syndrome trial. The cited MFI has no sleep domain; the reported “Sleep Quality Score” was not defined as a validated sleep measure or analyzed as a treatment contrast. This is fatigue/daytime-function evidence, not sleep efficacy.	If sleep is a target, use a validated, prespecified sleep instrument and analyze sleep separately from fatigue and daytime function.
Oleocanthal animal sleep [9,10]	Oral 10 mg/kg/day and a 0.5–20 mg/kg/day range; three months. Behavioral sleep changed; 20 mg/kg liver toxicity signal.	Male disease-model mice; inactivity-based sleep, not EEG architecture; no CBN.	EEG-confirmed sleep, relevant oral exposures, both sexes and repeated-dose safety.
CBN animal pharmacology [14,25]	Rat CBN 10–100 mg/kg IP; 10 mg/kg/day for 15 days. High-dose mouse repeated treatment produced tolerance/withdrawal; THC substitution.	Route/species mismatch; active-metabolite exposure uncertain in humans.	Oral parent/metabolite measurements and clinically relevant tolerance/impairment assessment.
Preliminary molecular/patent combinations [11,12,13]	CBN–phenolic TRPA1 cell assays; CBD–oleocanthal 1:8 hippocampal experiment. Supplementary Table S3 specifies provenance and available details.	Non-sleep assays; author-linked disclosures; parent-compound concentration relevance unresolved.	Replicate at measured exposures using individual components, antagonists and an explicit interaction model.
Formulation evidence [22,44,64]	Oil matrices affect cannabinoid handling/stability; rat CBD/THC exposure enhanced with lipids.	Carrier evidence, not a defined phenolic contribution or CBN-specific human superiority.	Separate carrier, phenolic and chemical-stability comparisons.

OleoMetS refers to the randomized olive-oil-phenol supplementation study in metabolic syndrome by Samoutis et al. [8], not a compound or a sleep trial. CBN, cannabinol; CBD, cannabidiol; THC, Δ9-tetrahydrocannabinol; EEG, electroencephalography; IP, intraperitoneal; TRPA1, transient receptor potential ankyrin 1. Numerical doses are observed research exposures, not treatment recommendations. Molecular findings and physicochemical formulation findings are intentionally separate categories.

## Data Availability

No new participant-level data were generated. The revision-stage search record and source-characterization tables are provided in the Supplementary Materials.

## Supplementary Materials

The following supporting information can be downloaded at https://www.mdpi.com/article/10.3390/nu18213659/s1: Table S1. Revision-stage source retrieval and selection record. Table S2. Material identity, exposure and purity reporting. Table S3. Non-sleep combination evidence: what is and is not specified.
