# Supplementary material for: Cannabinol for Sleep and Insomnia: A Critical Narrative Review and an Olive-Derived Polyphenol Combination Hypothesis
Source: Nutrients. 2026 Nov 4;18(21):3659. doi: 10.3390/nu18213659 (PMC13766135; doi:10.3390/nu18213659)
Supplement: Supplementary file 1 [file nutrients-18-03659-s001.zip › nutrients-4641731-supplementary.pdf]

Supplementary Methods and Evidence Tables

Manuscript: Cannabinol for Sleep and Insomnia: A Critical Narrative Review and an Olive-Derived Polyphenol Combination Hypothesis. Manuscript ID: nutrients-4641731. Reference numbers below refer to the revised main manuscript. This supplement distinguishes publicly verified details, unavailable source details and proposed future methods; no new experiments or participant-level analyses are reported.

Table S1. Revision-stage source retrieval and selection record

Scope: a targeted narrative update and correction exercise completed on 20 October 2026. The 64 references of the submitted manuscript were the defined seed bibliography. The submitted version documented a 21 September 2026 update with co-author-nominated material; its original database export and complete contemporaneous search log were not available for reconstruction. The present record does not retrospectively replace that history with a systematic-search claim.

S1A. Executed keyword searches and source interfaces.

Question	Executed search wording	Source domains / purpose
CBN and sleep	cannabinol sleep insomnia trial 2026	PubMed / PubMed Central indexed records via domain-focused web search; verify clinical studies and identify potentially relevant additional reports.
Olive phenolics, sleep and exposure	oleocanthal oleacein sleep fatigue plasma oleoglycine	PubMed and ScienceDirect records; parent/adduct exposure and clinical/preclinical endpoint specificity.
Combination mechanisms	cannabinol oleocanthal oleacein TRPA1 combination Dadiotis	ScienceDirect, Cell and Google Patents records; exact-title and publication-number follow-up.
CBN exposure and safety	cannabinol bioavailability oral pharmacokinetics melatonin tolerance purity	PubMed / PubMed Central records; oral exposure, metabolites, tolerance and interaction evidence.
Carrier study	Zgair dietary fats pharmaceutical lipid excipients increase systemic exposure 2016 doi	Primary publication record and full-text repository.
Oleacein mechanism	Wakasugi oleacein TrkB 2024 purity	Publisher article; binding, inhibitor studies and acute versus repeated in-vivo exposure.
Exact record checks	10.1016/j.drugalcdep.2026.113341; 10.1111/bcpt.14120; 10.1016/j.toxrep.2026.102296; WO2025219345A1	Publisher, PubMed, author-institution and patent records; title/DOI matching rather than generic keyword retrieval.
Regulatory / methods framework	SANRA narrative review; ICH Q2(R2), Q14, M10, Q1A(R2), Q1B, M3(R2), E6(R3); CTIS Regulation 536/2014; cannabinoids novel food; FDA cannabis regulation; Health Canada CBN	Primary method paper and official EMA, FDA, European Commission and Health Canada sources.

Date limits and language: no lower publication-date limit was applied; source inclusion stopped at 20 October 2026. English-language reports or records with interpretable English content were used. Domain-focused web retrieval is not identical to direct PubMed Boolean searching; results depend on indexing and are not an exhaustive MEDLINE, Embase, Scopus or trial-registry search. Retrieval totals and a PRISMA flow diagram are therefore not reported. Exact-title/DOI checking and backward citation follow-up supplemented the expressions above.

Inclusion: primary CBN sleep interventions; animal physiological sleep or relevant safety/exposure work; olive-compound sleep, sleep-loss or human fatigue studies; pharmacokinetic and formulation studies pertinent to oral translation; and directly relevant combination disclosures. Negative primary endpoints, next-day findings and toxicology were retained. Mixed-cannabinoid, dietary-observational and non-sleep models were labelled indirect. Exclusion from the clinical efficacy synthesis: unrelated disease applications, marketing assertions, duplicate reports and non-peer-reviewed records presented as if they were clinical efficacy studies. Conference and patent records were retained only in a separately qualified mechanistic category.

Appraisal and access: no independent duplicate screening, formal risk-of-bias score or quantitative pooling was performed. Some primary full texts, supplementary certificates and patent figure entries were inaccessible or incompletely rendered during revision. A verified abstract, published methods section, detailed reviewer correction and complete article are not interchangeable source-access levels. Missing purity or assay parameters are marked below rather than filled from marketing specifications. The updated synthesis does not claim that no unlocated combination work exists.

Table S2. Material identity, exposure and purity reporting

Material/exposure inventory for the most consequential studies.

Study / material	Model, route and duration	Composition / purity status	Interpretive boundary
Bonn-Miller [16]	Poor-sleep adults; oral 20 mg CBN alone or with CBD 10/20/100 mg; 7 nights.	Doses verified from the primary abstract. Numerical batch purity: NV.	Modified intention-to-treat N = 293; dose is not chemical purity.
Kolobaric [30]	Adults seeking better sleep; oral CBN 25/50/100 mg; 4 weeks.	Proprietary CBN preparation identified. Numerical trial-batch purity: NV in this source check.	Do not substitute a current commercial specification or patent claim for the trial batch.
Lavender / CUPID [17, 20]	Diagnosed-insomnia adults; oral 30/300 mg CBN; acute crossover.	Purified CBN preparation described; numerical administered-batch purity: NV.	Human exposure and performance effects are dose/formulation specific.
Arnold [14]	Male rats; IP CBN 10/30/100 mg/kg; repeated 10 mg/kg/day for 15 days; separate metabolite experiments.	Authors report 100% CBN purity with in-house LC–MS/MS assessment.	A reported analytical purity value is not proof that every possible impurity is absent; IP exposure is not oral human exposure.
Yang oleocanthal [9, 10]	Male Alzheimer's-model mice; oral 10 mg/kg/day and 0.5–20 mg/kg/day; 3 months.	Oleocanthal identity reported. Numerical study-specific purity not independently verified (NV).	Do not assign the preparation the purity of an unrelated isolate or equate oleocanthal with oleacein.
OleoMetS [8]	102 metabolic-syndrome adults; oral extract 10 mg/day; 12 weeks.	Approximately 80% combined oleocanthal/oleacein and 18% oleuropein/ligstroside aglycones.	These are mixture proportions, not purity or separate doses of each constituent.
Wakasugi oleacein [51]	SH-SY5Y cells, 1–50 μM for 24 h; acute oral reporter experiment and 10 mg/kg/day oral mouse inflammation study for 10 days.	Synthesis route cited in Methods. No numerical purity stated in the accessed main Methods.	TrkB binding/inhibitor inference; no direct receptor phosphorylation or sleep endpoint.
Vanegas CBN [25]	Mouse discrimination plus repeated high-dose treatment, including 100 mg/kg twice daily for 5 days.	Material is CBN; numerical batch purity: NV.	Behavioural tolerance/dependence endpoints cannot be negated by an unrelated general-toxicity study.
Bauter CBN isolate [26]	90-day oral rat toxicology; reported no-adverse-effect level up to 400 mg/kg/day.	Tested proprietary CBN isolate; numerical batch purity: NV.	Applies to that product and assay battery; not a combination or long-term human sleep safety study.
Dadiotis / patent combinations [11, 12, 13]	Acute cell/slice assays and separately reported non-sleep animal observations.	Exact batch purity and all concentration pairs not verified; see Table S3.	Concentrations, ratio claims and chemical purity must remain separate.

NV = numerical value not independently verified from source material accessible for this revision; NV does not mean the original report necessarily omitted it. IP = intraperitoneal; LC–MS/MS = liquid chromatography–tandem mass spectrometry; TrkB = tropomyosin receptor kinase B; CBN = cannabinol; CBD = cannabidiol. Authors should replace NV only after checking the actual study report/supplement or batch certificate; a supplier's present-day specification is insufficient.

Table S3. Non-sleep combination evidence: what is and is not specified

Combination records and source-level limits.

Record / model	Components and available exposure detail	Reported endpoint	What remains unresolved
WO2025219345A1, Example 1 / Figure 1 [13]<br>Transfected HEK293E cells expressing rat or human TRPA1.	CBN with oleocanthal or oleacein is included. Acute addition after 30 s; response sampled for a further 150 s. Exact individual/combination concentrations and batch purity require original figure verification.	Calcium mobilization; wild-type and Y840A receptor comparisons.	HTML unit corruption and embedded figure tables prevent reliable transcription of every concentration. General claimed ratios (1:3–1:40) are not substituted for experiment-specific doses.
WO2025219345A1, hippocampal experiments / Figures 9–10 [13]	CBD plus oleocanthal; reported 1:8 combination, with HC-030031 antagonist comparison; acute perfusion in high-potassium conditions.	Suppression of spontaneous hyperexcitable activity; antagonist attenuated the effect.	CBD is not CBN. Concentration-unit typography and exact exposure duration should be verified against the original document before adding numerical µM values. No sleep outcome.
Dadiotis conference communication, Phytochemistry Letters [11]	Cannabinoid–olive-secoiridoid combinations. Available conference-level material does not adequately establish the cannabinoid identity/ratio for the cited single-dose mouse observation.	Preliminary molecular/non-sleep observations.	Underlying methods/datasets beyond public disclosure are not included. No inferred repeated-dose combination safety.
Dadiotis conference abstract, Biophysical Journal [12]	CBD and oleocanthal; TRPA1 pharmacological/mechanistic experiments.	Preliminary channel/network interaction report.	Not CBN-specific sleep evidence. Exact concentration–response, replication and material details require the complete abstract/source data.
Other patent experiments [13]	Multicomponent combinations containing CBN in cancer-cell systems, including 48-hour exposure assays.	Non-sleep cellular activity.	These cannot identify the effect of CBN plus one phenolic on physiological sleep or qualify repeated oral use. Not used as clinical efficacy evidence.

CBN = cannabinol; CBD = cannabidiol; TRPA1 = transient receptor potential ankyrin 1. The patent is a published application, not a peer-reviewed clinical report or marketing authorization. Evangelos Dadiotis is a named inventor; Ekati Biotech PC is the listed applicant. No experiment-specific concentration is inferred from a broad claim ratio or from a corrupted unit in machine-readable patent text.

Interpretation notes

Kolobaric multiplicity: the revision reports longitudinal placebo-relative p values as nominal and notes that they would fail a conservative Bonferroni correction across three CBN–placebo contrasts. This statement is deliberately distinct from asserting that the same correction was prospectively required for that exact testing family. The full article, supplement and original statistical plan should be reconciled before describing the scope of a prespecified adjustment. No reanalysis of participant data was conducted.

Lavender safety: the revised event distribution and next-day task-specific corrections follow the detailed study information identified during peer review. Event totals are not participant incidence rates. The primary WASO estimates are reported with confidence intervals, and no generalized impairment of every driving endpoint is claimed. Original result tables should be available to the author team for final numerical reconciliation.

Graphical abstract: supplied separately. It summarizes the distinction between observed CBN/olive-phenolic findings and the unconfirmed clinical-combination hypothesis. The two main figures are embedded in the manuscript and supplied separately in vector SVG and high-resolution PNG formats.
